# Estimation of rocks’ failure parameters from drilling data by using artificial neural network

**DOI:** 10.1038/s41598-023-30092-2

**Published:** 2023-02-23

**Authors:** Osama Siddig, Ahmed Farid Ibrahim, Salaheldin Elkatatny

**Affiliations:** 1 Petroleum Engineering Department, King Fahd University of Petroleum and Minerals, Dhahran, 31261 Saudi Arabia; 2 Center for Integrative Petroleum Research, King Fahd University of Petroleum and Minerals, Dhahran, Saudi Arabia

**Keywords:** Engineering, Civil engineering, Mechanical engineering

## Abstract

Comprehensive and precise knowledge about rocks' mechanical properties facilitate the drilling performance optimization, and hydraulic fracturing design and reduces the risk of wellbore-related problems. This paper is concerned with the failure parameters, namely, cohesion and friction angle which are conventionally estimated using Mohr's cycles that are drawn using compressional tests on rock samples. The availability, continuity and representability, and cost of acquiring those samples are major concerns. The objective of this paper is to investigate an alternative technique to estimate these parameters from the drilling data. In this work, more than 2200 data points were used to develop and test the correlations built by the artificial neural network. Each data point comprises the failure parameters and five drilling records that are available instantaneously in drilling rigs such as rate of penetration, weight on bit, and torque. The data were grouped into three datasets, training, testing, and validation with a corresponding percentage of 60/20/20, the former two sets were utilized in the models' building while the last one was hidden as a final check afterward. The models were optimized and evaluated using the correlation coefficient (R) and average absolute percentage error (AAPE). In general, the two models yielded good fits with the actual values. The friction angle model yielded R values around 0.86 and AAPE values around 4% for the three datasets. While the model for cohesion resulted in R values around 0.89 and APPE values around 6%. The equation and the parameters of those models are reported in the paper. These results show the ability of in-situ and instantaneous rock mechanical properties estimation with good reliability and at no additional costs.

## Introduction

### Rock failure parameters

In oil fields, many downhole problems such as borehole instability and sand production are directly related to the rock’s mechanical properties^1^. Hence, a good knowledge of the rock's mechanical characteristics can help to minimize the problems during the drilling operation and can be used to optimize the drilling performance and enhance the economic gain from reservoir^1,2^. Furthermore, the determination of the geomechanical properties of the reservoir and near-reservoir rocks is important for the hydraulic fracturing design, and reservoir/geomechanical modeling^3^.

Friction angle and cohesion are important geomechanical properties that reflect the shearing strength, the angle of rupture and the stability condition of the materials^4,5^. These parameters are essential when conducting the stability analysis^6,7^. Cohesion and friction angle are affected by many factors such as particle arrangement, material physical properties, and loading conditions. Cohesion reflects the internal force that bonds the material’s particles together while friction angle reflects the frictional resistance within the material^8^.

Mohr–Coulomb criterion is frequently used for rock failure characterization, in which shear stress (τ) is assumed to have a linear relationship with the effective normal stress (σ′) as per (Eq. 1^9^). Where the intercept is known as cohesion (C) in stress units, also called inherent shear strength, and the slope is the tangent of the angle of internal friction (φ) in degrees, also called friction angle.

1 $$ \uptau = \text{C} + \tan \left( \upvarphi \right)\upsigma^{^{\prime}} $$

**Figure 1:**
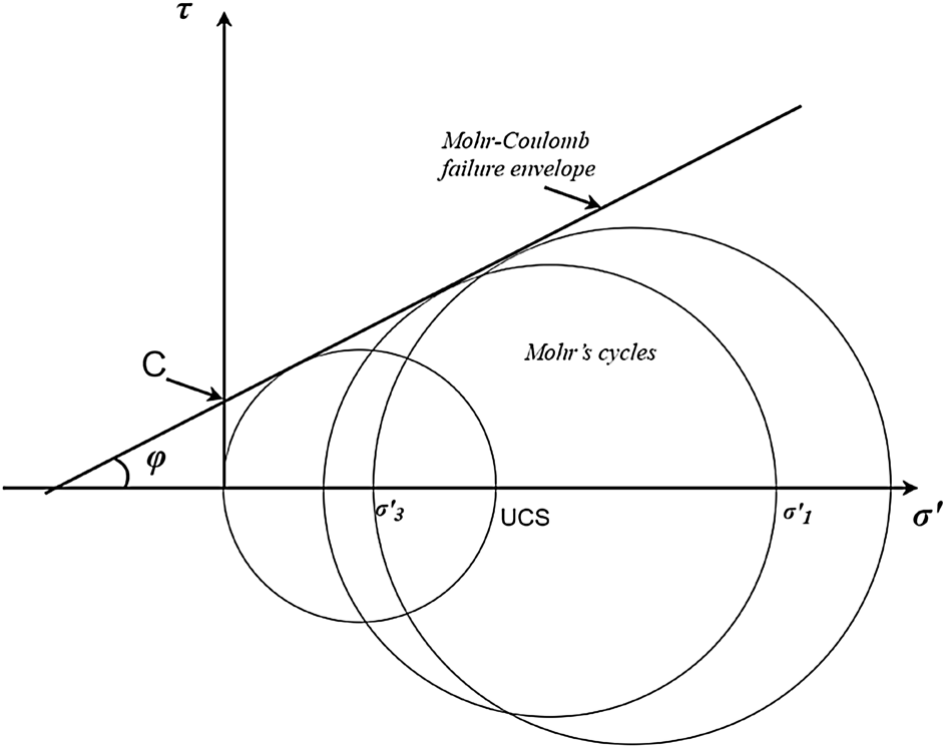
Estimation of failure parameter from Mohr’s cycles.

Estimation of the rock failure parameter usually requires several compressional tests to draw multiple Mohr’s cycles and then the Mohr–Coulomb failure envelope can be drawn as a tangent for those cycles^10,11^. The angle between the failure envelope and the normal stress axis is the friction angle and the intersection with the shear stress axis is the cohesion as in Fig. 1. In this way, C and φ describe how rock fails under different horizontal stresses.

This process of estimating the failure parameters is costly and time-consuming, furthermore, the availability and the representability of the core samples are major concerns, in addition to that, it is difficult provide continuous information due to the number of samples limitation. Holt et al.^12,13^ pointed out that the extracted core samples exerted mechanical properties change due to the release of the stresses. Many researchers tried to overcome these concerns by correlating the failure parameters to other physical rock properties that are easier to be measured.

### Cohesion and friction angle correlations

Several attempts to correlate φ and C with the porosity (Ø) have been made, it has been reported that both parameters decrease with porosity, however, the accuracies of these correlations are low with R^2^ values didn’t exceed 0.76^1,14,15^. The correlations are expressed with linear relation as in the following (Eq. 2 to Eq. 5):

Weingarten & Perkins:

2 $$ \upvarphi = 57.8 - 1.05\emptyset $$

Edimann et al.:

3 $$ \upvarphi = 41.929 - 0.7779\emptyset $$

4 $$ \text{C} = 37.715 - 0.8757\emptyset $$

Abbas et al.

5 $$ \varphi = 64.369 - 99.238\emptyset $$

Plumb^16^ and Chang et al.^17^ incorporated the effect of shale content in φ estimation using the gamma-ray (GR) (Eq. 6 and Eq. 8), the former reported an increase of φ with clay content. Almalikee^18^ also reported a correlation between GR and φ expressed by (Eq. 9).

Plumb:

6 $$ \upvarphi = 26.5 - 37.4\left( {1 - \emptyset - V_{\text{shale}} } \right) + 62.1\left( {1 - \emptyset - V_{\text{shale}} } \right)^{2} $$

where V_shale_ is calculated by (Eq. 7):

7 $$ V_{\text{shale}} = \frac{{\text{GR - GR}_{\min } }}{{\text{GR}_{\max } - \text{GR}_{\min } }} $$

Chang et al.:

8 $$ \upvarphi = \tan^{ - 1} \left( {\frac{{\left( {\text{GR - GR}_{sand} } \right)\mu_{\text{shale}} + \left( {\text{GR}_{\text{shale}} - \text{GR}} \right)\mu_{sand} }}{{GR_{shale} - GR_{sand} }}} \right) $$

where GR_sand_ and GR_shale_ are the gamma-rays of pure sand and shale respectively which were reported to be 60 API and 120 API with in same order by the original authors. μ_shale_ and μ_sand_ are the internal friction coefficients (tanφ) for pure shale and sand respectively (reported to be 0.5 and 0.9 respectively by the authors).

Almalikee:

9 $$ \upvarphi = 39.25 - 0.1166\,\text{GR} $$

In addition to the porosity and GR, φ and C have been correlated to the compressional wave velocity (V_p_), both parameters increase with V_p_ as in (Eq. 10 to Eq. 12^19,20^).

Lal:

10 $$ \upvarphi =  \sin ^{{ - 1}} \left( {\frac{{{\text{V}}_{{\text{p}}}  - 1}}{{{\text{V}}_{{\text{p}}}  + 1}}} \right)  $$

11 $$ C = \frac{{5\left( {\text{V}_{\text{p}} - 1} \right)}}{{\sqrt {\text{V}_{\text{p}} } }} $$

Abbas et al.:

12 $$ \varphi = 17.134e^{{0.239 \text{V}_{\text{p}} }} $$

The efforts toward obtaining empirical correlations for the failure parameters were not limited to the above equations, several authors employed machine learning (ML) techniques for the same objectives. The applications of ML in the estimation of rock’s physical and mechanical properties are growing due to its high accuracy. These applications cover but are not limited to the correlations of porosity^21,22^, permeability^23,24^, bulk density^25^, compressive strength^26^, sonic velocities^27^ and elastic properties^28^. Cohesion and friction angle were not an exception, different authors presented ML-based estimations for them. In addition to the porosity, Vp, and GR, the models’ inputs include shear wave velocity (V_s_) and bulk density (ρ_bulk_) as summarized in Table 1.

**Table 1:** Summary of machine learning models for cohesion and friction angle.

Ref	Inputs	ML method/s	R	No. of data points
The angle of internal friction				
Alloush et al.^29^	Ø, GR, ρ_bulk_, V_p_, V_s_	ANN, ANFIS, SVM	0.87–0.99	353
Tariq et al.^30^	Ø, ρ_bulk_, V_p_, V_s_	ANFIS, SVM, FN	0.81–0.92	120
Hiba et al.^31^	Ø, ρ_bulk_, V_p_	ANN	0.98	1900
Cohesion				
Tariq et al.^30^	Ø, ρ_bulk_, V_p_, V_s_	ANFIS, SVM, FN	0.81–0.93	120
Hiba et al.^31^	Ø, ρ_bulk_, V_p_	ANN	0.97	1900

### Utilization of drilling data

All the models in Table 1 require, at least, the knowledge of sonic wave velocities, bulk density and porosity. Therefore, the failure parameters cannot be estimated unless we have these inputs that need a well logging operation. As an alternative, this work proposes using drilling data instead of well logs. The advantages of drilling parameters over the well logging outcomes are that the former requires no additional cost and is easier to be obtained and available at an earlier stage in the life of the well.

In the oil industry, one of the oldest exploitations of the drilling operational data is in the estimation of the formation pressure. Recently, employing machine learning, the drilling data were utilized in the prediction of rock properties such as bulk density^25^, wave velocities^32^, static and dynamic Young’s modulus^33^, static and dynamic Poisson’s ratio^34,35^.

The objective of this paper is to present an investigation on the use of drilling data in the prediction of rock failure parameters utilizing the artificial neural network as a machine learning tool. The advantage of drilling data over the tests on core plugs is that the drilling data are available at an earlier stage, more frequent, and require no additional cost. Therefore, this approach will help in having an instantaneous and complete profile for those parameters which should be very beneficial for the optimization of drilling and fracturing operations.

## Methodology

**Figure 2:**
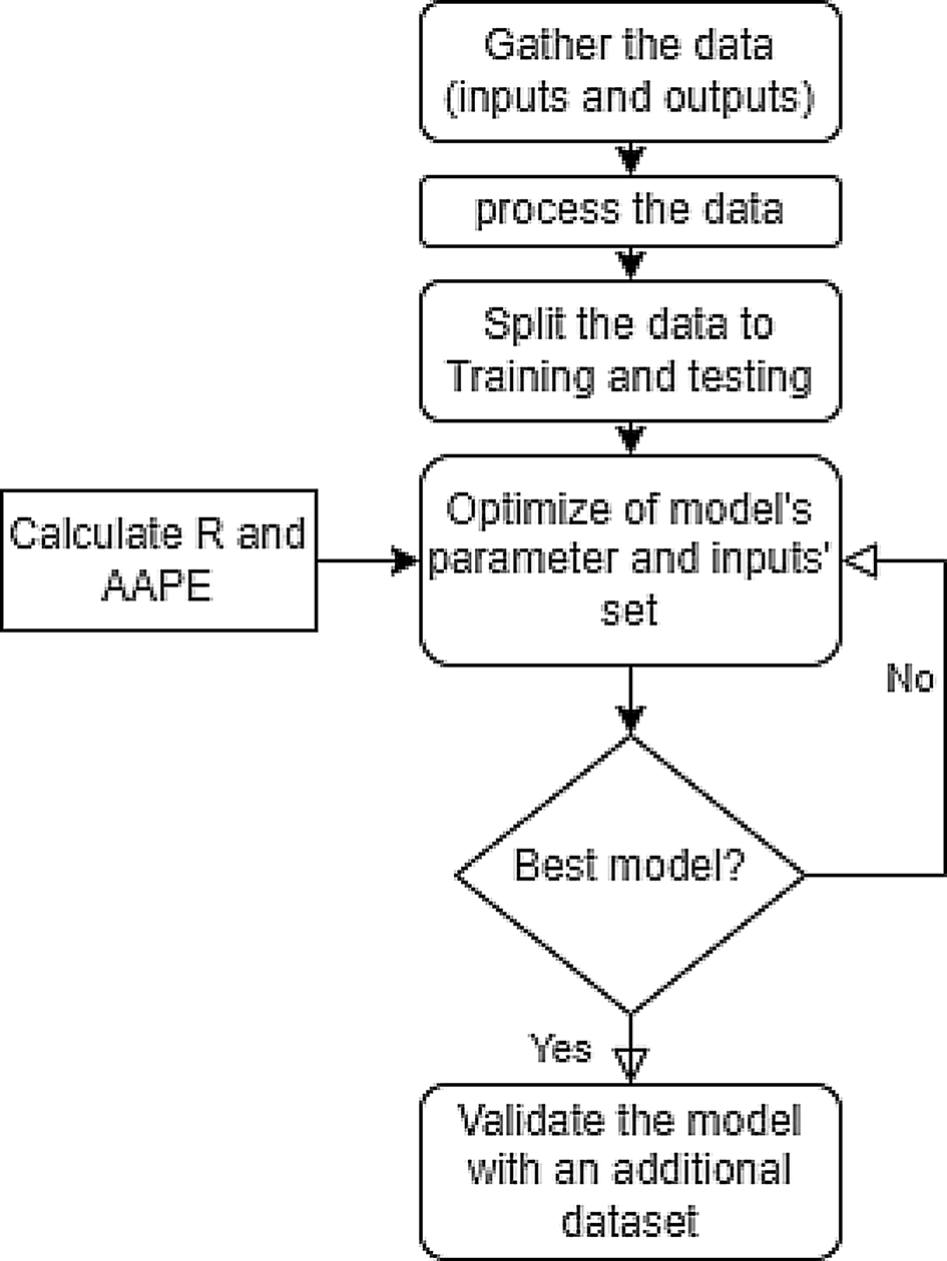
Research methodology flowchart.

The following procedure, illustrated in Fig. 2, has been employed to predict failure properties from the drilling parameters. Data for drilling operation records and experimental tests have been compiled and divided into three groups after the preprocessing. In the pre-processing step the different parameters were normalized using min–max normalization method (parameter value-minimum value)/(maximum value − minimum value) to scale the parameters to varies between 0 and 1. The different equations used to normalize the different parameters, in addition to recalculating the output data from the normalized values are listed in the Appendix A. According to their accuracies, the models are updated and optimized to provide the best possible performance.

### Data

The utilized dataset contains over 2200 data points, the data has been divided into three groups and different division percentages were tested (from 50:25:25 to 80:10:10). The best outcomes were notices with 60:20:20 percentage of data division; following are the definitions of these data groups and percentages. 60% of the data were used to train the model (Training dataset), and 20% of the points were used to test the model accuracy within the algorithm to update the model’s parameters (Testing dataset). The last 20% of the dataset was hidden from the machine learning tool to validate the built models (Validation dataset). The definitions of these terms (training, testing and validation), may be different in some other publications. For instance, in some literature, the validation dataset refers to the dataset provided along with the training, and the testing dataset is the final evaluation. However, in this paper, the former definitions are maintained.

Each measurement contains five drilling history recordings as inputs, as well as values for cohesion and friction angle that are established as the targeted outputs. This model was built using the following drilling parameters acquired from field data:

- Drilling rate of penetration ROP
- Weight on bit WOB
- Drill pipe pressure SPP
- Torque
- Drilling fluid pumping rate

**Figure 3:**
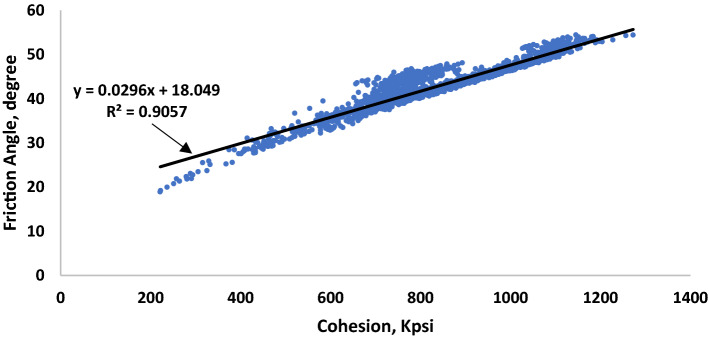
The correlation between cohesion and friction angle.

The data were cleaned of noise and abnormalities using the Matlab program before being entered into the ANN. Table 2 presents the statistical analysis of the three datasets, the three datasets cover slightly different ranges of inputs and outputs parameters with an average change in the mean values of 11%. The lowest relative standard deviation values were noticed for Q, SPP and T ranged between 0.04 and 0.14 while the highest values were for ROP between 0.5 and 0.57. The linear correlation coefficient values between the five input values and the two output values were all less than 0.58 which indicates that there are no direct linear relationship between each input individually and each output. However, data shows a high correlation coefficient between the two failure parameters as seen in Fig. 3. The models presented in the results section of this paper will be limited by the ranges presented in Table 2.

**Table 2:** Statistical parameters for the training data.

	Q (gpm)	SPP (psi)	T (kft.lbf)	WOB (klbf)	ROP (ft/h)	C (1000 psi)	Friction Angle (degree)
Training							
Minimum	192	1749	2.60	5.78	3.20	221	18.91
Mean	241	2668	2.98	9.21	24.53	816	42.35
Maximum	271	3152	3.73	14.90	65.03	1197	53.96
Relative standard deviation	0.09	0.12	0.06	0.22	0.50	0.19	0.12
R with C	0.10	0.11	0.17	− 0.13	0.01	1.00	0.94
R with φ	− 0.11	− 0.08	0.17	− 0.12	0.00	0.94	1.00
Testing							
Minimum	192	1809	2.64	5.78	4.80	412	28.52
Mean	239	2909	3.31	13.28	17.35	920	45.19
Maximum	270	3152	3.72	14.90	63.17	1173	53.42
Relative standard deviation	0.08	0.14	0.08	0.27	0.57	0.15	0.09
R with C	0.51	0.55	0.29	0.59	− 0.50	1.00	0.92
R with φ	0.25	0.27	0.05	0.44	− 0.43	0.92	1.00
Validation							
Minimum	236	2517	2.70	5.90	3.53	281	21.82
Mean	249	2884	3.11	10.61	20.41	877	43.53
Maximum	271	3153	3.73	14.45	60.44	1157	53.43
Relative standard deviation	0.04	0.07	0.09	0.30	0.55	0.19	0.12
R with C	− 0.22	0.15	0.35	0.25	− 0.18	1.00	0.99
R with φ	− 0.20	0.14	0.32	0.25	− 0.19	0.99	1.00

### Machine learning

**Figure 4:**
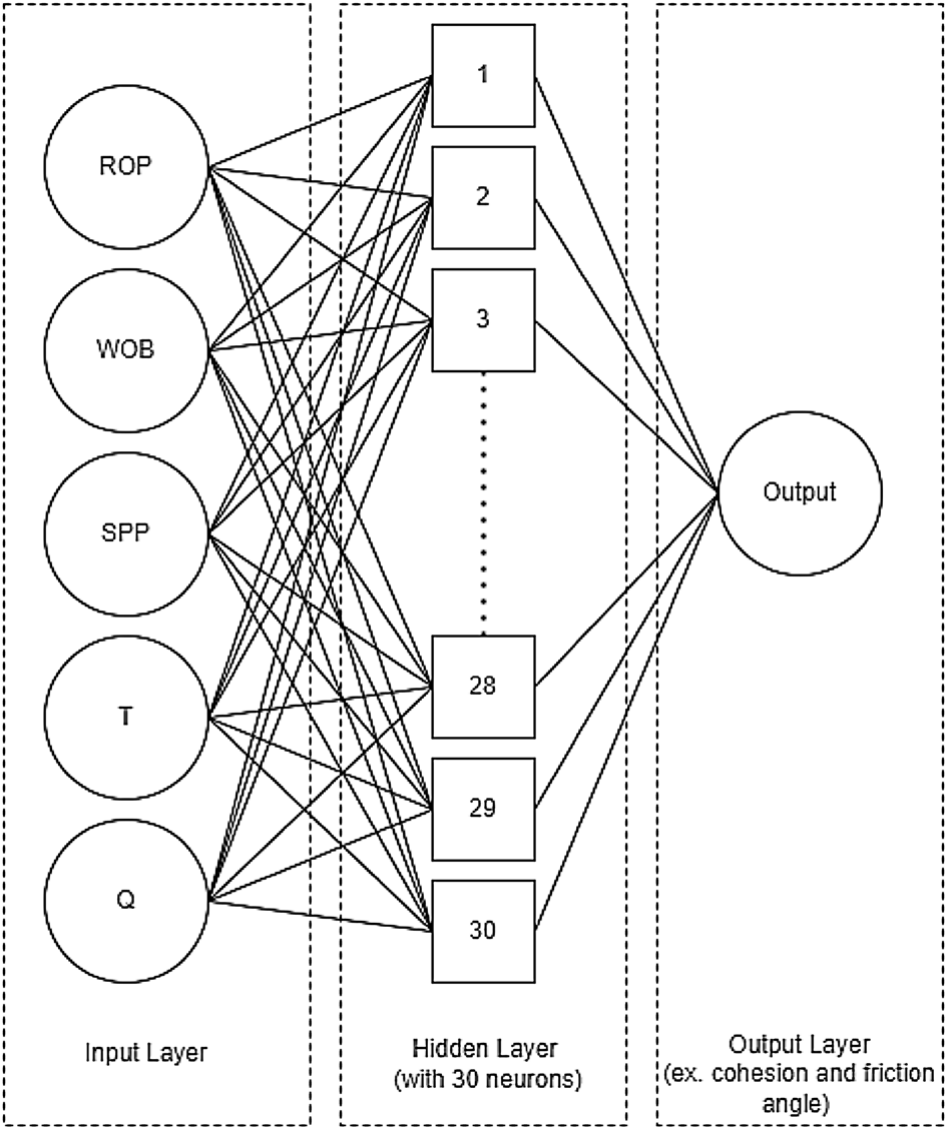
Structure of the artificial neural network.

Artificial neural networks (ANN) were used to build empirical correlations between cohesion/friction angle and drilling parameters. ANN is a popular machine-learning method that simulates brain neurons^36^. In classification, regression, and clustering tasks, ANN could be used as an unsupervised or supervised machine learning tool^37^. As shown in Fig. 4, an ANN is made up of several elements such as neurons, training functions, and transfer functions in different layers^38^. Many effective applications of ANN in the oil and gas industry have been reported in the literature^24,39^. For instance, ANN has been utilized successfully in developing correlations for porosity^40^, permeability^41^, drilling fluid rheology^42^, rate of penetration^43^, and hydrocarbon properties^44^.

In this work, the Bayesian regularization backpropagation method was utilized for training the network and updating weight and bias values based on Levenberg–Marquardt optimization. A logistic sigmoid was used as the activation function to calculate the required outputs. Ascending numbers of neurons were tested and stopped when no further significant improvements were noticed, the 30 neurons in Fig. 4 were given as an example.

### Model evaluation

Different runs were performed in the ANN to determine the optimum tuning elements within the algorithms. The number of neurons and the types of employed training/network/transfer functions were all evaluated. All of these models' trials were evaluated using two statistical measures: the correlation coefficient (R) and the average absolute percentage error (AAPE) which have been calculated using (Eqs. 13 and 14), respectively:

13 $$ R = \frac{{\left[ {N\mathop \sum \nolimits_{i = 1}^{N} \left( {X_{given i} \times X_{{{\text{Predicted}} i}} } \right)} \right] - \left[ {\mathop \sum \nolimits_{i = 1}^{N} X_{given i} \times \mathop \sum \nolimits_{i = 1}^{N} X_{{{\text{Predicted}} i}} } \right]}}{{\sqrt {\left[ {N\mathop \sum \nolimits_{i = 1}^{N} \left( {X_{given i} } \right)^{2} - \left( {\mathop \sum \nolimits_{i = 1}^{N} X_{given i} } \right)^{2} } \right]\left[ {N\mathop \sum \nolimits_{i = 1}^{N} \left( {X_{{{\text{Predicted}} i}} } \right)^{2} - \left( {\mathop \sum \nolimits_{i = 1}^{N} X_{{{\text{Predicted}} i}} } \right)^{2} } \right]} }} $$

14 $$ AAPE = \frac{{\mathop \sum \nolimits_{i = 1}^{N} \frac{{X_{given i} - X_{Predicted i} }}{{X_{given i} }} \times 100\% }}{N} $$

where N is the size of the dataset, $$X_{given}$$ and $$X_{Predicted} $$ are respectively the measured and the ANN-estimated failure parameter values.

## Results and discussion

### Training and testing

The models were optimized to yield the best possible fitting accuracy in terms of the higher value of R and the lower value of AAPE. The best performance was found Bayesian regularization backpropagation training function and log-sigmoid transfer function. The maximum number of epochs was set at 2000, however the optimum performance was found at 836, and 1412 epoch in the case of cohesion, and friction angle models, respectively.

**Figure 5:**
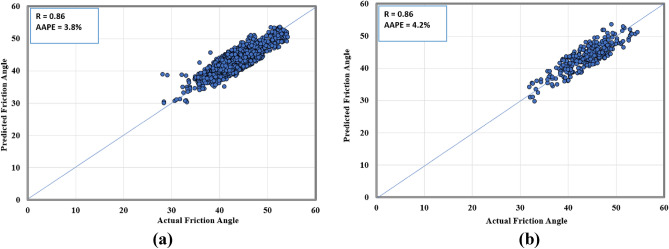
Actual versus predicted friction angle cross plots for (**a**) training and (**b**) testing datasets.

**Figure 6:**
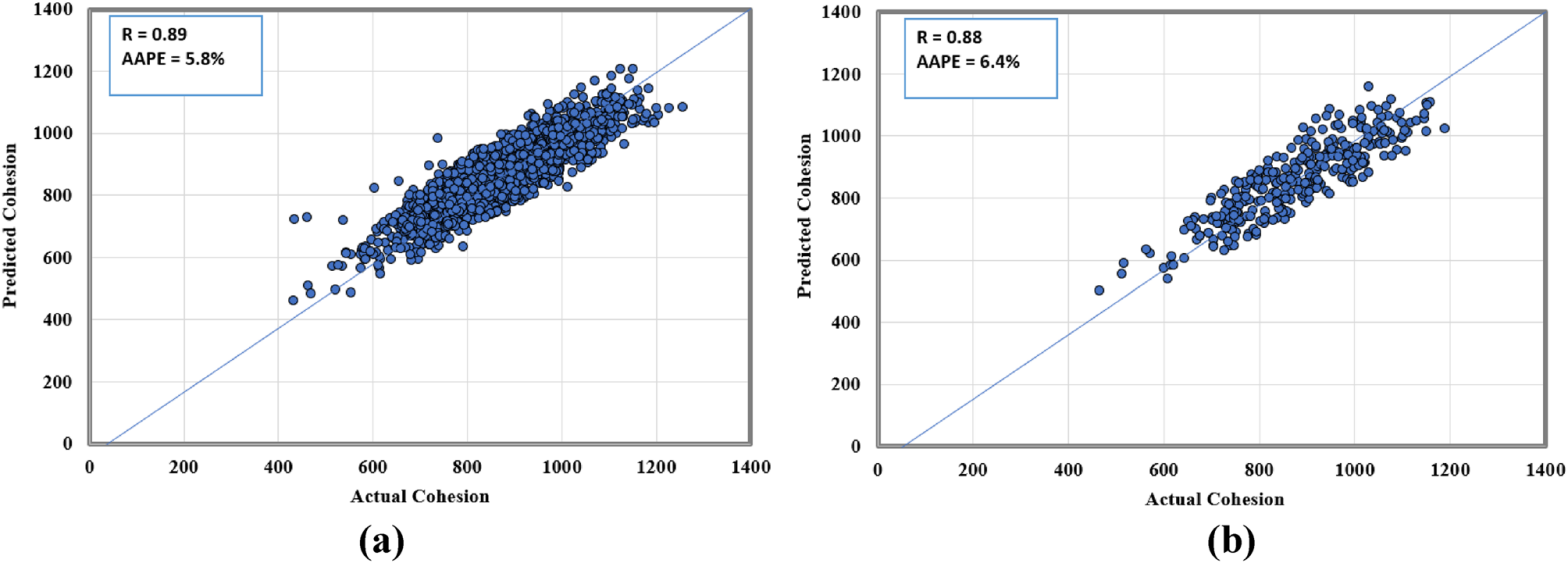
Actual versus predicted cohesion cross plots for (**a**) training and (**b**) testing datasets.

Figures 5, 6 show the cross plots between the actual and estimated failure parameters for the training and the testing. The closer the points are to the 45-degree line means better the prediction. For the friction angle, the model resulted in a 0.86 correlation coefficient for both training and testing, while the AAPE values were around 4% ± 0.2%. Similarly, the resulting R values for cohesion ranged between 0.88 and 0.89 and AAPE values were in the range between 5.8 and 6.4%. A similar performance in predicting the two parameters was expected since they have a high correlation coefficient as shown in Table 2 and Fig. 3.

### Models’ validation

**Figure 7:**
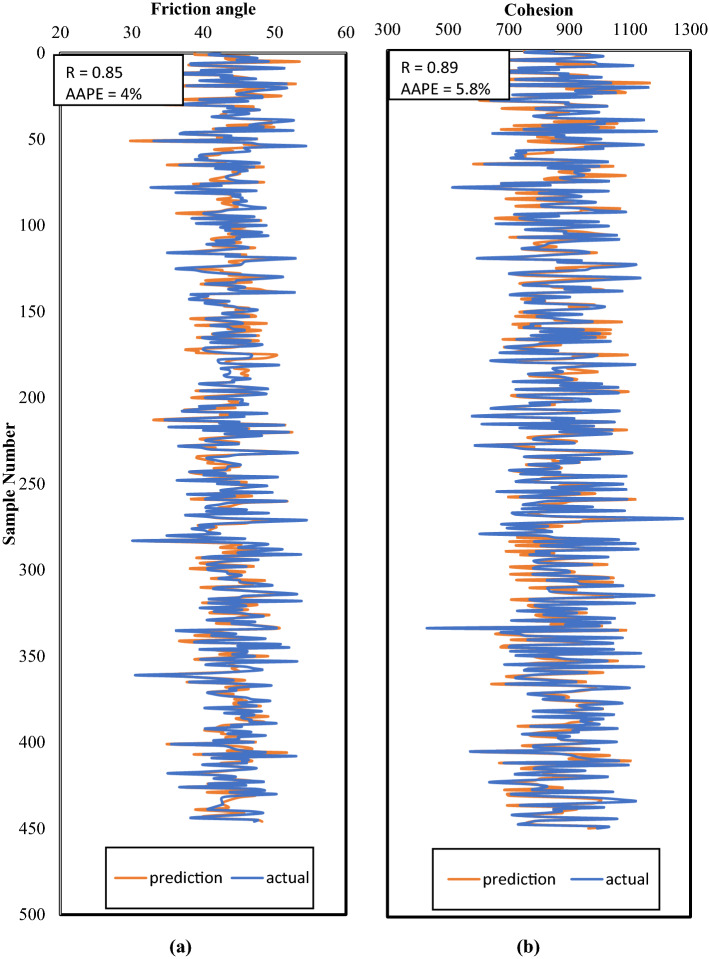
Comparison between the actual and predicted profiles for validation dataset for (**a**) friction angle and (**b**) cohesion.

Figure 7 shows a visual comparison between the actual and estimated values using the constructed models on the validation dataset. The performance of the models in the validation was very similar in accuracy to the training and testing, for instance, validation R values were 0.85 and 0.89 for friction angle and cohesion respectively, compared to 0.86 and 0.89 in the same order for the training. Similarly, the validation AAPE values were 4% and 5.8% for φ and C respectively, and the values in the same order were 3.8% and 5.8% for the training. Those results in validation confirm a good generalization of the model for the investigated data range.

**Figure 8:**
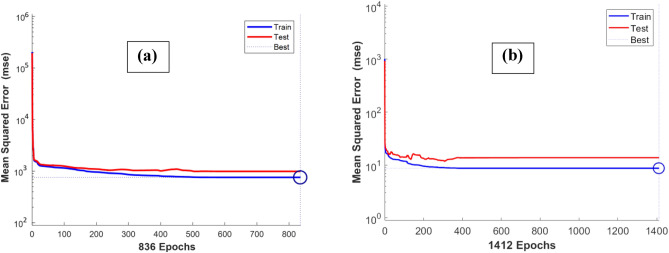
Training performance in terms of MSE showing the best performance at (**a**) epoch 836 for cohesion model, and (**b**) epoch 1412 for friction angle model.

By comparing the current method, which resulted in correlation coefficients in the range between 0.86 and 0.89, as in Figs. 5, 6, 7, with previous attempts based on machine learning mentioned in Table 1, which resulted in correlation coefficients in the range between 0.81 and 0.99, the results are close with a different input in both cases. Deducing the properties of rock failure using drilling data gives some positive advantages over using well logs data as in previous models, which are that drilling data is always available and before any other data in the well and does not need additional cost and at the same time it can provide continuous information because it is recorded in a frequent and real-time manner. It worth mentioning that the network training performance is shown in Fig. 8 in which the MSE was used as the loss function to monitor the model performance. It is clear from Fig. 8 that overfitting issue did not occur while running the model.

### Models’ equations

The best models were achieved using the log-sigmoid transfer function and 30 neurons in the ANN. Equation (15) and Eq. (17) present the model for cohesion and friction angle respectively. While Table 3 and Table 4 present the models' parameters needed for Eq.(15)and Eq. (16) respectively. Using these equations and the data in tables allows them to be tested in different datasets to generate synthetic failure parameters or to be compared with any model that would be built in the future with similar parameters.

15 $$ C_{n} = \left[ {\mathop \sum \limits_{i = 1}^{N} W_{2,i} \left( {\frac{1}{{1 + e^{{ - \left( {W_{11,i} *Qn + W_{12,i} *SPPn + W_{13,i} *Tn + W_{14,i} *WOBn + W_{15,i} *ROPn + b_{1,i} } \right)}} }}} \right)} \right] + b_{2} $$

**Table 3:** The parameters in Eq. (15) for cohesion estimation.

i	W11	W12	W13	W14	W15	W2	b1	b2
1	− 0.4626	3.9477	0.3820	− 3.7261	− 2.7384	4.5612	− 3.0487	0.7534
2	− 1.7512	0.3995	− 0.9573	− 1.8493	− 3.4812	3.6093	− 2.9639	
3	3.4467	− 2.6912	− 2.0344	1.3955	− 2.3160	− 4.0910	1.3472	
4	− 3.3115	1.7078	4.7183	5.1277	2.3778	− 2.3839	6.4843	
5	− 0.6096	3.5467	2.1475	− 1.8022	− 3.9018	3.0889	− 5.0166	
6	0.8459	0.1030	− 3.4423	− 4.0818	− 0.7629	3.1857	− 1.7508	
7	0.5604	− 3.5778	1.7044	− 2.2264	− 2.1836	− 3.4758	− 2.8909	
8	− 0.1979	− 1.4184	2.8321	0.3343	4.5631	3.7736	− 1.2807	
9	− 3.4217	3.5302	− 3.4351	4.5828	− 6.1035	− 2.6635	− 0.2858	
10	− 4.2223	2.7503	4.2267	− 0.8829	3.5359	− 2.7252	− 1.6227	
11	3.9493	3.0739	0.3628	− 3.9052	− 2.3685	− 3.9817	− 2.5630	
12	− 4.2528	1.6032	− 3.4292	3.6714	− 1.5528	− 2.6967	1.2954	
13	2.1650	− 1.1716	3.8909	0.7559	− 2.0511	5.9139	4.6457	
14	4.7619	3.4076	0.9669	− 0.3775	− 1.4343	− 5.3146	− 2.5483	
15	0.9869	6.1320	0.7224	− 2.9455	− 1.6232	− 5.5960	1.1852	
16	5.2747	2.0909	− 5.1466	5.5499	4.9855	− 1.9117	1.4404	
17	3.7714	6.0683	− 1.5366	2.6079	1.2224	2.7830	− 3.3447	
18	− 0.9427	− 0.0848	3.2698	2.5618	8.5606	1.8185	2.7887	
19	− 4.6161	3.7344	− 0.3284	− 0.1666	1.2752	− 5.2042	− 0.3783	
20	0.2343	− 0.2793	− 2.8602	− 3.2140	− 2.4216	3.9275	2.1866	
21	1.3340	− 0.3624	− 1.1518	− 2.5195	− 5.1351	− 5.3687	− 4.8506	
22	5.5159	2.9003	− 0.5477	3.7368	1.3668	5.8258	− 5.0941	
23	− 1.3679	4.0048	6.0178	0.1877	− 3.3167	1.6285	0.7768	
24	− 2.9959	3.2193	− 0.6533	3.8584	− 1.9523	3.3315	3.4024	
25	− 1.3169	− 0.3113	4.0184	4.7077	− 2.3668	− 3.3486	5.8567	
26	− 7.8940	− 4.3963	0.4776	− 0.4482	− 0.0418	5.0919	5.7940	
27	− 4.0545	0.3185	2.4356	− 4.3961	− 4.1931	− 3.6754	− 1.9184	
28	− 1.5350	− 2.0027	− 1.1113	3.5188	− 0.7597	− 3.3823	− 1.4046	
29	− 1.2392	1.4004	− 1.9070	2.6803	− 3.0978	7.9737	0.2013	
30	− 6.1130	− 1.5863	− 2.5822	− 1.6123	1.0860	− 3.5113	0.7738

**Table 4:** The parameters in Eq. (16) for friction angle estimation.

i	W11	W12	W13	W14	W15	W2	b1	b2
1	− 2.7268	3.1729	− 1.8966	1.4533	− 3.3696	3.9083	− 2.3290	0.4914
2	1.0234	− 2.7712	− 1.1594	− 1.5927	− 6.9518	2.7626	1.2426	
3	− 2.2030	2.1522	2.7034	1.0662	2.2571	− 2.5175	− 0.9233	
4	8.4448	1.6358	− 2.4581	1.7105	0.7717	− 4.7423	− 4.0064	
5	1.0771	− 3.1006	− 3.4333	− 1.6010	0.5316	− 4.9676	− 3.3831	
6	3.6045	0.0828	1.0656	− 1.0406	− 3.1155	− 4.1456	− 1.5837	
7	− 5.4181	− 0.1021	− 0.5423	3.7557	0.5413	− 2.7456	− 2.2564	
8	1.5684	− 1.1616	− 0.7748	− 2.0457	− 5.4494	3.6664	− 2.7260	
9	1.5636	1.0480	− 1.6678	4.2732	− 2.0324	− 4.0835	− 2.1537	
10	3.0395	4.6627	− 3.1102	4.1239	− 5.3634	2.2452	1.0630	
11	2.2964	− 0.1194	− 2.2336	− 3.7195	2.9661	2.0912	0.7943	
12	− 6.5031	4.1496	3.1487	− 2.9225	− 1.2062	− 4.2507	0.6373	
13	4.2289	0.3285	1.0464	1.1993	3.0443	− 5.5712	0.6665	
14	3.1476	4.5677	− 2.6159	− 0.3767	− 1.5617	− 5.5610	1.6434	
15	− 0.1853	0.7083	− 4.8352	1.5455	1.0554	4.1587	0.6326	
16	− 0.7986	2.9249	2.1390	3.0546	5.8311	2.4651	1.0338	
17	5.8532	− 5.0631	3.9285	− 4.6260	3.0886	1.7535	0.8709	
18	0.6294	− 1.4129	− 1.0115	− 3.3946	− 6.3743	− 2.3799	− 5.0854	
19	− 1.5265	4.3705	3.5004	− 3.9963	0.0326	3.8307	− 0.2961	
20	3.7355	− 0.2991	7.5053	− 4.3698	2.8003	1.9072	0.9474	
21	− 0.4720	2.8605	− 3.0669	− 2.9864	2.0164	2.6845	− 4.4890	
22	4.3245	0.2441	1.9603	0.6809	5.7271	2.3403	1.0564	
23	1.6552	2.0243	5.1369	1.0186	− 3.4166	3.0640	− 3.1808	
24	− 2.4345	− 4.7504	4.2931	− 2.7364	− 2.0916	3.0464	1.3291	
25	− 1.0378	− 2.5454	− 4.5141	3.6022	0.2019	− 2.9869	3.2390	
26	1.7275	− 2.8544	− 4.8205	− 5.3222	− 1.2669	1.6206	− 5.9244	
27	− 0.7008	− 1.0796	2.7139	1.3403	2.5573	2.7815	1.3251	
28	− 6.1850	2.4880	0.9860	− 1.2513	1.5460	− 5.0449	1.5920	
29	− 5.0047	3.5075	− 0.0028	− 1.9242	− 0.4709	− 5.0888	− 4.7672	
30	− 1.8034	− 0.2083	− 3.6769	3.5196	− 1.8058	5.8040	− 0.3378	
31	− 0.7123	− 4.0905	0.7652	− 4.1395	3.3275	3.2667	− 1.2940	
32	− 6.2928	− 1.2435	1.9389	− 3.3260	− 4.8744	− 2.3704	0.0551	
33	1.2263	− 3.4571	− 1.3846	− 2.4011	− 2.1300	− 3.4178	2.8852	
34	3.0520	6.3290	− 2.7741	3.4476	2.1349	2.2798	− 2.3840	
35	0.9507	2.6073	6.5060	2.2539	− 2.8463	2.5637	1.8638	

The normalized cohesion value can be back transformed to the actual value using the following equation.

16 $$ C = 976 C_{n} + 221 $$

17 $$ \varphi_{n} = \left[ {\mathop \sum \limits_{i = 1}^{N} W_{2,i} \left( {\frac{1}{{1 + e^{{ - \left( {W_{11,i} *Qn + W_{12,i} *SPPn + W_{13,i} *Tn + W_{14,i} *WOBn + W_{15,i} *ROPn + b_{1,i} } \right)}} }}} \right)} \right] + b_{2} $$

The normalized friction angle value can be back transformed to the actual value using the following equation.

18 $$ \varphi = 35.05 \varphi_{n} + 18.91 $$

It should be highlighted that the application of the developed correlations in equations. (15) and (16) to predict the friction angle and cohesion are more recommended for carbonate formations from which the data used in developing the models were obtained. Therefore, some errors might be expected upon the application for different formation lithology. Moreover, it is recommended to employ the developed equations using inputs within the range and the same units listed in Table 2 to ensure reliable results.

## Conclusions

Rock mechanical parameters are vital in drilling optimization, fracturing design, and avoiding borehole problems. Conventionally, rock failure parameters are estimated using the Mohr–Coulomb failure envelope that requires drawing multiple Mohr's cycles and hence performing several compressional tests on rock samples. In this paper, an alternative technique based on the utilization of drilling data and artificial neural network is investigated and presented with the following concluding remarks:

- The proposed approach has an advantage over the experimental testing or the previous ML-based models that require well logging data; because the drilling data are available earlier than the well logs and their acquisition does not require additional operational cost. In addition, in contrast to core samples which have practical limitations in the number of samples that could be obtained, drilling data can provide continuous information.
- The models for the two parameters yielded close performance in all datasets, training, testing, and validation, even though the last one was not introduced during the models’ building.
- For friction angle, the yielded R values were around 0.85 and 0.86 while AAPE values were between 3.8 and 4.2% for the three datasets.
- For cohesion, the model resulted in R values between 0.88 and 0.89 and AAPE values ranged between 5.8 and 6.4%.
- The comparable matching accuracy in the two parameters could be attributed to the observed high correlation coefficient between the two failure parameters.
- In previous works, rock bulk density and elastic properties have been predicted from drilling data, in addition to the failure properties presented in this paper. For future work, the same approach could be applied to estimate other properties such as petrophysical properties.

## Supplementary Information

Supplementary Information.

## Data Availability

The datasets generated and/or analyzed during the current study are not publicly available due to confidentiality but are available from the corresponding author on reasonable request.
