# Supplementary material for: Estimation of rocks’ failure parameters from drilling data by using artificial neural network
Source: Sci Rep. 2023 Feb 23;13:3146. doi: 10.1038/s41598-023-30092-2 (PMC9950081; doi:10.1038/s41598-023-30092-2)
Supplement: Supplementary file 1 — Supplementary Information. [file 41598_2023_30092_MOESM1_ESM.doc]

**Appendix A**

The input and output parameters were normalized using the following equations:

$Q_{n}=0.01266 \left( Q-192 \right)$ (A-1)

${SPP}_{n}=0.00071 \left( SPP-1749 \right)$ (A-2)

$T_{n}=0.88496 \left( T-2.60 \right)$ (A-3)

${WOB}_{n}=0.10965 \left( WOB-5.78 \right)$ (A-4)

${ROP}_{n}=0.01617 \left( ROP-3.20 \right)$ (A-5)

$C_{n}=0.001024 \left( C-221 \right)$ (A-6)

$\varphi_{n}=0.02853 \left( \varphi-18.91 \right)$ (A-7)

The subscript "n" refers to the normalized form of the input and out parameters.
